# Respiratory and airway disorders in children with Down Syndrome: a review of the clinical challenges and management

**DOI:** 10.3389/fped.2025.1553984

**Published:** 2025-03-13

**Authors:** V. E. Craven, W. J. Daw, J. W. Y. Wan, H. E. Elphick

**Affiliations:** Department of Respiratory Medicine, Sheffield Children’s Hospital, Sheffield, United Kingdom

**Keywords:** Down Syndrome, airway, sleep disordered breathing, immunodeficiency, GORD

## Abstract

Down Syndrome (DS), or Trisomy 21, is a common inherited chromosomal disorder, caused by an extra copy of chromosome 21, with features including intellectual disability, hearing and vision disorders, hypotonia, hypothyroidism, cardiac and gastrointestinal structural abnormalities. The characteristic features of flattened nasal bridge, mandibular and maxillary hypoplasia, relative macroglossia, and a narrow nasopharyngeal region all predispose to airway complications and structural abnormalities can extend to the lower airways and lung parenchyma. Congenital airway stenoses and malacia are present in around 1.5% children with DS and in 20% of these, there are multiple anomalies. Structural lung abnormalities include reduced alveolar numbers and altered lung architecture. The prevalence of pulmonary hypertension is a significantly increased, estimated to affect 5-10%, and increases if congenital or gastrointestinal co-morbidities are also present. The association of DS with hypotonia, increased oral secretions, gastrointestinal reflux and aspiration and obesity increase the morbidity associated with these anatomical variants contributing to poor airway clearance and increased risk of respiratory tract infections. In addition, it is been recognised that the increased risk of infections (particularly of the respiratory tract) as well as autoimmune disorders and haematological malignancies suggest a level of immunodeficiency and immune dysregulation. The anatomical features of DS predispose children to the development of sleep disordered breathing (SDB) in addition to adenotonsillar hypertrophy, the primary cause in children. Treatment options include surgery, non-invasive ventilation, and anti-inflammatory medications. Emerging techniques include drug-induced sleep endoscopy (DISE), a useful tool for assessment of the upper airway in children with OSA and to identify the additional sites of airway obstruction that may be present in DS and hypoglossal nerve stimulation for individuals resistant other treatments.

## Introduction

Down Syndrome (DS), or Trisomy 21, is a common inherited chromosomal disorder, caused by an extra copy of chromosome 21, with an incidence of approximately 1 in 800 births (1). The common features of individuals with DS, first described by John Langdon Down in 1866 (2), are delayed psychomotor development, intellectual disability, and hypotonia. Their characteristic physical features include brachycephaly, a flattened nasal bridge, mandibular and maxillary hypoplasia, relative macroglossia, and a narrow nasopharyngeal region (3). The typical medical comorbidities include congenital defects of the heart, gastrointestinal tract abnormalities such as duodenal atresia and tracheoesophageal fistula, airway abnormalities, such as laryngomalacia and tracheomalacia, coeliac disease, hearing and vision disorders, hypothyroidism, obesity and obstructive sleep apnoea (OSA) (4). In this article we will review the respiratory and airway co-morbidities associated with DS, highlighting the current gaps in the literature and suggesting future directions for research.

## Structural airway abnormalities

Structural airway abnormalities are common in Down Syndrome (DS) and its association with hypotonia, increased oral secretions and obesity increase the morbidity associated with these anatomical variants. An Australian cohort study of 405 children with DS found that admissions secondary to congenital airway abnormalities occurred in 1.5% of this population and accounted for 2.5% of all their admissions at a median age of 1 year (5). Bronchoscopy in children with DS with chronic respiratory symptoms yielded anatomical abnormalities in 71% compared to 32% in an otherwise healthy control group presenting with similar symptoms (6). Upper and lower airway endoscopy is therefore important in the investigation of chronic respiratory symptoms in children with DS.

### Upper airway obstruction due to anatomical variants

Narrowing of the nares and flattening of the nasal bridge can cause nasal obstruction. Although tongue size is normal, relative macroglossia due to a small mouth and oropharynx is common. Patients often have midfacial hypoplasia and whilst adenoids and tonsils may be normal in size or enlarged, crowding can occur and can cause upper airway obstruction. Lingual tonsil hypertrophy is also ten times more common in children with DS (7). All these anatomical variants can cause symptoms of upper airway obstruction.

### Laryngomalacia

Within the first 2 years of life, laryngomalacia is the commonest cause of upper airway obstruction, becoming less prevalent after this age. Laryngomalacia is an anomaly of the larynx causing an inward collapse of the supraglottic airway during inspiration. It typically worsens with feeding, crying and lying supine, it can present as stridor, poor feeding, coughing, vomiting and respiratory distress. In severe cases, poor feeding and/or an increased work of breathing can result in failure to thrive.

Laryngomalacia was found in 50% of patients with DS undergoing bronchoscopy (8). It is most commonly diagnosed in children with DS suspected to have airway obstruction in the neonatal period (9). It can be managed conservatively if mild, but in severe cases, supraglottoplasty is indicated. Other airway abnormalities such as tracheomalacia or subglottic stenosis, should also be excluded.

65%–100% of infants with laryngomalacia have gastroesophageal reflux disease (GORD) (10), and this is particularly common in DS. The airway obstruction caused by laryngomalacia generates a negative intrathoracic pressure which can cause reflux. This exposes the laryngeal tissue to acid which in turn causes oedema, exacerbating tissue collapse. Treatment for GORD with positioning, feed thickeners and a proton pump inhibitor should be considered in the management of laryngomalacia in DS.

### Acquired tracheal stenosis

Children with DS have smaller tracheal diameters and therefore require smaller calibre endotracheal tubes, at least two sizes smaller than would be determined using standard anaesthetic formulae. Age has been found to be the more reliable factor when choosing an endotracheal tube. An MRI study of DS children without respiratory symptoms showed a tracheal diameter 1.3–3.2 mm smaller compared to children without DS (11). This discrepancy leads to a greater incidence of tracheal trauma upon intubation and a resultant iatrogenic subglottic stenosis.

Subglottic stenosis is a narrowing of the upper airway between the vocal cords and the proximal trachea. The subglottis is the narrowest section of the airway and any further narrowing can significantly affect airflow leading to persistent stridor, recurrent croup and in severe cases, respiratory distress. 33% of children with DS were reported to have a post-extubation stridor, far greater than in children without DS (12).

### Congenital tracheal stenosis

Congenital subglottic stenosis is much less common than acquired subglottic stenosis. It affects about 1.4% of the DS population (8). Complete tracheal rings that lack a posterior membranous wall can cause mid or distal tracheal stenosis ranging from a few centimetres to the entire tracheal length. They typically cause an hourglass stenosis that might be incidentally found during an elective intubation or if more severe can present with stridor, dyspnoea, or respiratory distress,

Complete tracheal rings are associated with congenital heart disease such as ventricular or atrial septal defects and Tetralogy of Fallot and so an echocardiogram is indicated.

### Management of tracheal stenosis

Mild tracheal stenosis with less severe symptoms and shorter segments can be managed conservatively as airway growth with age can resolve symptoms. In subglottic stenosis, balloon dilatation can be performed but this might need to be carried out repeatedly. In more severe cases, management includes open laryngotracheal reconstruction with tracheal resection and an end-to-end anastomosis or slide tracheoplasty. Complete tracheal rings may continue to grow after resection (13) and surgical recovery may be more complicated in DS with co-morbidity. In the most severe cases, tracheostomy may be necessary.

### Tracheal bronchus

A tracheal bronchus is an aberrant or additional bronchus arising from the trachea, the majority arising on the right sides. In isolation the significance of a tracheal bronchus remains controversial although cases of recurrent right upper lobe pneumonia have been described. Bertrand et al. described a tracheal bronchus in 21% vs. 2.1% of non-DS controls all of whom were undergoing a flexible bronchoscopy for respiratory symptoms (8).

### Tracheal compression and congenital heart disease

40% of children with DS have congenital heart disease (CHD). Vascular anomalies can cause a fixed, pulsatile compression of the trachea and children can present with stridor, chronic cough or biphasic wheeze, the severity of which is related to the position and extent of the compression. Despite the high prevalence of CHD in DS, vascular anomalies causing significant symptoms are relatively uncommon. An anomalous right subclavian artery was found in 6% of children with DS, increasing to 25% of those being investigated with a flexible bronchoscopy for respiratory symptoms (14).

The most common vascular anomaly is a double aortic arch which encircles both the trachea and the oesophagus causing respiratory and gastrointestinal symptoms, but other vascular abnormalities include anomalous innominate artery, right aortic arch with an aberrant left subclavian artery and a pulmonary artery sling. Airway compression can also occur as a result of left to rights shunts such as ventricular septal defects, atrial septal defects and patent ductus arteriosus.

Compression on an airway causes malacia, on that segment. Bertrand et al. described congenital heart disease occurring in 63% of those with DS and tracheobronchomalacia (8).

### Tracheobronchomalacia

Airway malacia is the collapse of a lumen during expiration. It can occur in isolation due to poor cartilaginous development or be secondary to compression by a vascular anomaly or CHD. It can affect the trachea (tracheomalacia), the bronchi (bronchomalacia) or both (tracheobronchomalacia). It can present with chronic cough, dyspnoea, or recurrent infections due to its effect on secretion clearance. In severe cases it can present with failure to extubate, or sudden collapse. Malacia is reported in up to 7.1% of children with DS (15) and in up to 46% of children with DS undergoing bronchoscopy for respiratory symptoms (6) although it is likely that is underestimated.

### Multiple airway anomalies

Multiple airway anomalies are highly prevalent in DS, accounting for around 20% of the airway anomalies, compared to 5% in comparable controls. If one airway anomaly is found it is prudent to look for the coexistence of others and consider other contributing pathology from other systems.

## Structural lung abnormalities

**Figure 1:**
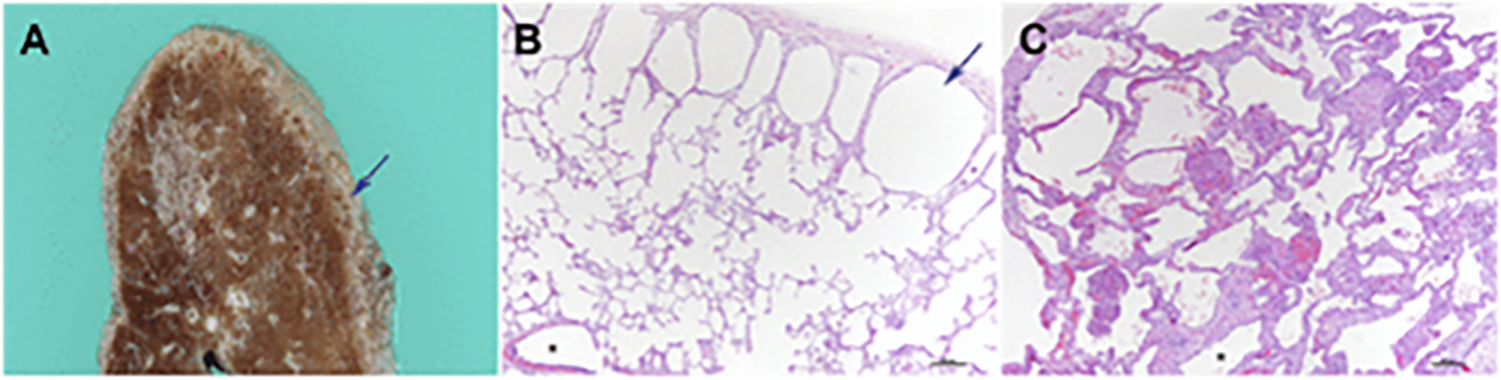
Adapted from Danopoulos et al. 2021 (with permission). Lung pathology in DS. **(A**,**B)** subpleural cystic dilatation of alveolar spaces **(C)** pulmonary hypoplasia in an 8-month old infant with congenital heart disease is visualized on lung biopsy by the markedly enlarged alveolar spaces, compared with alveolar spaces in **(B)**. Reference: Danopoulos S. et al. Lung disease manifestations in Down syndrome Am J Physiol Lung Cell Mol Physiol 321: L892–L899, 2021.

Children with DS are prone to structural lung abnormalities, including reduced alveolar numbers, and altered lung architecture (16). These structural anomalies, combined with hypotonia can contribute to poor airway clearance and increased risk of respiratory tract infections. Figure 1 illustrates the lung disease manifestations in DS.

The lungs of children with DS are often observed as being hypoplastic. The lung parenchyma has a diffuse porous pattern, attributed to a 58%–83% decrease in alveolar numbers and enlarged alveolar airspaces (17) and up to a 25% reduction in branch generation (18). Consequently, there is a diminished lung surface area for gaseous exchange and reduced functional reserve (19), further exacerbating respiratory compromise in these children. Subpleural cysts are also frequently seen in children with DS (20). These are benign, small cystic dilatations along the lung's subpleural surface, typically measuring 1–2 mm in diameter. Although rarely visible on plain chest radiograph, they can be detected on CT scans. One study reviewing DS children with prior CT imaging of the lungs reported their presence in 36% of children (20). Despite their frequency, they generally hold no clinical significance and do not seem to provoke respiratory issues or pulmonary hypertension.

Children with Down Syndrome also exhibit anomalies in alveolar vasculature, including an immature double capillary network (21) with increased intercapillary spacing and a higher incidence of intrapulmonary anastomoses (22). These structural differences may contribute to the heightened severity of pulmonary hemosiderosis and isolated pulmonary capillaritis observed in this population (23). The lymphatic endothelium may also exhibit abnormalities including lymphatic hypoplasia, dilation, and dysfunctional lymphangiogenesis (24). These alterations can result in impaired lymphatic drainage and the development of conditions such as pulmonary lymphangiectasia and chylothorax (24). Children with Down Syndrome face a unique set of challenges related to structural lung abnormalities that can significantly impact their respiratory health. Innovations in imaging techniques, such as lung MRI and ultra-low-dose CT, are likely to improve the ability to detect and monitor these. Whether routine imaging is required is still up for debate but delineating the degree of hypoplasia and identifying other structural anomalies may allow for better prognostication and tailored management improving future outcomes.

### Pulmonary hypertension

Pulmonary hypertension (PHT) is a significant condition in children with Down Syndrome (6). Infants and children with DS face a heightened risk of PHT, with prevalence rates reported as high as 28% (25). In newborns with DS, PHT is estimated to affect 5%–10% (26). PHT is often multifactorial. It may result from congenital heart disease with left to right shunt lesions causing excess pulmonary blood flow and intrinsic endothelial dysfunction (27). However, PHT can also arise independently of structural cardiac abnormalities, often associated with comorbidities like upper airway obstruction, swallowing dysfunction, gastroesophageal reflux disease, and aspiration, leading to intermittent hypoxia and lung inflammation (26).

Early diagnosis and monitoring of PHT in children with DS is crucial given the progressive nature of the condition. There are, however, no accepted screening guidelines with screening mainly limited to comorbid conditions (28). Echocardiography is often the first-line tool for screening and can be used as a regular monitoring tool. However, if diagnosis is uncertain cardiac catheterisation may be indicated but is invasive and not always feasible (29).

A few noteworthy studies have explored potential biomarkers for PHT in children DS. Colvin et al. (30) identified higher circulating levels of fibrocytes and myeloid-derived suppressor cells in six children with DS and pulmonary arterial hypertension, suggesting a potential link between these cellular markers and disease progression. Similarly, Bush et al. (31) found that children with DS exhibited elevated endostatin levels but reduced angiogenin levels, with these altered peptide levels being more frequently associated with the presence of PHT. This led to the hypothesis that dysregulated angiogenesis, driven by changes in circulating peptides, might contribute to the development of PHT in this population. Though both studies were small in sample size, they offer important insights into the utility of biomarkers for identifying children with DS who may be at higher risk for pulmonary hypertension. These findings underscore the need for larger, more robust studies to validate the role of these biomarkers in clinical practice.

### Pulmonary function testing

The assessment of pulmonary function is a standardised objective tool that can be very useful in establishing baseline pulmonary function in DS. It can also be used for screening, detecting pathology and monitoring the effects of medical treatment or rehabilitation. Spirometry is inexpensive and widely available, however, cognitive impairment in DS can limit its use and care needs to be taken to ensure that it is done properly and that results are accurate (32). Given the difficulties of performing spirometry there is limited data, however, forced expiratory volume in 1 s (FEV_1_), forced vital capacity (FVC) and peak expiratory flow (PEF) are all lower in DS and lung function has been shown to be inversely proportional to body mass index (BMI) (33).

Oscillometry is an effort independent method of measuring airways resistance and reactance. This is a little studied area; however, it can be more successfully performed than spirometry (32). A study of 66 infants demonstrated a fixed obstructive picture at this early age with mildly increased lung volumes and significantly restricted expiratory flow rates that were unresponsive to bronchodilators (34).

## Sleep disordered breathing

Sleep disordered breathing (SDB) is used to describe a group of disorders characterised by abnormal respiratory patterns or insufficient ventilation during sleep. The most common type of SDB is obstructive sleep apnoea (OSA), characterized by snoring and repetitive episodes of upper airway obstruction during sleep, leading to sleep disturbance and/or abnormalities in gas exchange (hypoxia/hypercapnia) (35). Long-term consequences of OSA in children include pulmonary hypertension due to chronic nocturnal hypoxia and cognitive and behavioural impairment such as inattention and reduced academic performance due to repeated sleep disturbance (36).

DS is associated with several anatomical features that predispose children to the development of OSA such as mid-facial and mandibular hypoplasia, narrow nasopharynx, shortened palate, and relative macroglossia (due to crowding of the oropharynx), laryngomalacia. Subglottic and tracheal stenosis contribute towards an increased risk of SDB, in addition to adenotonsillar hypertrophy (7, 37). Other common co-morbidities of DS such as generalized hypotonia, an immature immune system (leading to more respiratory infections), a propensity for obesity, mucopolysaccharide deposition due to hypothyroidism and gastro-oesophageal reflux are all further risk factors in the pathogenesis and evolution of SDB in patients with DS (38–40).

As a result, OSA occurs with an increased prevalence of 31% and 79% (41–44), in contrast to the 1%–5% prevalence observed in the general paediatric population (45). Accurate prevalence figures are difficult to ascertain due to differences in patient selection criteria, definitions and methodologies used (46). Lee et al. reviewed 18 studies that included 1,200 children with mean age: 7.7 years; 56% boys and mean sample size of 67 patients (47). For children who underwent polysomnography, the prevalence of OSA based on AHI > 1, 5, and 10 events/h were 69%, 50%, and 34%, respectively. Meta-regression showed that AHI > 5 events/h was inversely correlated with age (*P* < .001) and younger age is associated with more severe disease (41) but a resurgence of symptoms often occurs with the onset of obesity in adolescent years, with an estimated risk ratio of 2.4 (48).

### Clinical impact

Children with DS are vulnerable to adverse complications of OSA, particularly those with congenital heart disease putting them at increased risk of developing pulmonary hypertension (42). Cognitive and behavioural sequelae are likely to be more problematic in DS children who have reduced cognitive reserve (49). Early detection and treatment of SDB in children with DS is therefore important.

#### Cognitive and behavioural function

There is substantial evidence regarding the negative impact of SDB on cognition and behaviour in healthy children (50–52). It is hypothesized that these adverse outcomes are mediated by the repetitive hypoxia and sleep disruption that are associated with SDB (50). Children with DS may be at greater risk due to sleep problems receiving less attention with priority being given to coexisting health issues (38) and an existing intellectual disability and behavioural phenotype leading to reduced recognition that SDB may be exacerbating these factors.

Two studies have demonstrated impairments in cognition, particularly verbal IQ, verbal fluency, and cognitive flexibility in children with DS and OSA compared to those who do not have OSA (53, 54). Breslin et al. found a verbal IQ of 9 points lower in children with DS and coexisting OSA on PSG compared with those without OSA (54) using a previously validated cognitive test designed specifically for use in children with DS. This study did not, however, find poorer attention in those with OSA compared to those without.

Churchill et al. provide strong evidence to suggest that sleep disturbances are negatively associated with the accomplishment of daily activities in children with DS, undertaking the largest community-based study to date involving 110 children with DS (55). The authors suggest that improvement in sleep problems such as sleep-disordered breathing could potentially translate to significantly improved quality of life (QOL) in children with DS.

Joyce and Dimitriou (56) showed that typically developing (TD) children with higher apnoea-hypopnoea indices had worse behavioural scores, but the same relationship was not seen in children with DS. Interestingly, total sleep duration was longer in the children with DS than the TD children and the authors hypothesized that this could have been protective, improving behaviour and cognitive scores for this group. This is in keeping with the findings of Brooks *et al*, who did not find a difference in behaviour between school-age children with DS with and without OSA but did demonstrate a positive relationship between total sleep time, percentage of slow-wave sleep, and cognitive function (57).

Nixon et al. 2016 studied the impact of OSA severity on adaptive functioning, a measure of how well a person handles common demands in life and how independent they are compared to others of a similar age and background in a cohort of 30 children of whom five (17%) had primary snoring, 7 (23%) had mild OSA, and the remaining 18 (60%) had moderate–severe OSA (58). Some associations were found between objectively assessed severity of OSA and adaptive functioning in children with DS, particularly in the area of communication skills.

Betavani et al. identified that treatment of SDB improves severity of the disease as defined by PSG, and this was associated with parental reports of improved, despite treatment having no demonstrable impacts on sleep quality, behaviour, or daytime functioning (59). Further, studies are needed to determine whether improvements in OSA seen after treatment in DS are mirrored by improvements in these key areas of daytime functioning. If so, OSA could be seen as a modifiable risk factor for poor functioning in children with DS.

#### Cardio-respiratory complications

OSA has been shown to be associated with cardiovascular sequelae such as hypertension (46) and left ventricular hypertrophy (60) in typically developing (TD) children. Given the increased incidence of OSA in DS, these children can be considered to be at high risk for cardiovascular complications and pulmonary hypertension occurs at a much higher rate in children with DS with or without CHD (61).

O’Driscoll et al. demonstrated that children with DS and SDB exhibit a compromised acute cardio-respiratory response. This is demonstrated by significantly reduced heart rate changes and delayed reoxygenation post obstructive event and increased time to re-saturation, alongside a dampened sympathetic response, demonstrated by significantly reduced overnight urinary catecholamines, compared with TD children with SDB (62). These data may reflect autonomic dysfunction, in children with DS that may place them at increased risk for cardiovascular complications such as pulmonary hypertension. Early detection and treatment of SDB may therefore be necessary to minimize cardiovascular risk in children with DS.

In support of this idea, relief of airway obstruction by intubation in children with DS has been shown to reverse pulmonary hypertension (63) Furthermore, case reports of children with DS and cor pulmonale have described improvement in cardiovascular status following surgical treatment of OSA (64, 65) It is also possible that children with DS are more likely to desaturate with apnoea as a result of reduced functional residual capacity associated with structural respiratory abnormalities.

### Diagnosis and screening

Clinical history and examination are poor predictors of PSG-diagnosed OSA in typically developing children (66, 67) and in children with DS (68). Routine screening has been recommended in some countries but is not standard practice. The American Academy of Pediatrics recommends referring all children with DS for a polysomnography by the age of 4 years (69). The British Thoracic Society guidance recommends annual screening from infancy until 3–5 years old with cardio-respiratory sleep studies (CRSS) for children with DS (70).

The cost, difficulty and limited availability of sleep study technology has led to the investigation of simpler screening methods. Sanders et al. developed a screening questionnaire using existing validated sleep questionnaire items to screen children with Down Syndrome up to 6 years of age for obstructive sleep apnoea but concluded that even a well-designed questionnaire with good psychometric properties had limited predictive value and could not be recommended for screening for moderate to severe OSA in young children with DS (71, 72).

Hanna et al. conducted a systematic review to identify predictors of SDB in patients with Down Syndrome. Inconsistent associations were found between a variety of variables and SDB in children with Down Syndrome, and the quality of evidence was poor. Meta-analysis identified only that children with OSA were older than those without OSA, suggesting a need for longitudinal screening to diagnose children who may develop SDB as they get older (73).

Hill et al. studied the value of home pulse oximetry and concluded that using the delta 12s index for screening could halve the number of children with Down Syndrome needing multichannel sleep studies and reduce the burden on children, families and health services (74).

### Treatment

#### ENT procedures

Adenotonsillectomy remains the first-line treatment option in children with DS but is acknowledged to be less successful than in TD children, with approximately 50% of children continuing to have residual OSA post-surgery, likely secondary to their higher rates of multilevel upper airway collapse (75, 76). Commonly identified sites of obstruction include the lingual tonsils, tongue base and the supraglottis and additional surgical procedures, including uvulopalatopharyngoplasty, lingual tonsillectomy, supraglottoplasty, partial midline glossectomy, and tongue suspension with or without lingual tonsillectomy have been suggested as surgical options, but there is currently limited evidence to support the routine use of these procedures (77).

Drug-induced sleep endoscopy (DISE) can be a useful tool for assessment of the upper airway in children with OSA (78) and to identify additional sites of airway obstruction that may be amenable to surgical interventions which may be performed under the same anaesthesia. Controversy remains as to how well DISE simulates physiological sleep and although it holds promise as a beneficial tool for children with DS a larger prospective study is needed before specific recommendations may be made on incorporating DISE into the OSA diagnostic and treatment algorithm for children with DS (79).

#### Non-invasive ventilation

Non-invasive ventilation (NIV) or continuous positive airway pressure (CPAP) therapy are often attempted for the management of persisting OSA or those not amenable to surgical procedures. Evidence to document effectiveness of ventilation and clinical outcomes is limited.

A review article by Hudson et al. identified 28 articles that included 543 children with Down Syndrome using long-term non-invasive ventilation (80). The review of this literature indicates that children with DS may have greater difficulty and take longer to initiate long-term NIV (81), with a higher rate of inability to establish use. Outcome data for those that do achieve establishment of NIV are similar to those for other children and suggest a favourable impact on cardiac function and attention with over half tolerating the respiratory support (82). The review was limited in its conclusions due to a high risk of bias associated with mostly retrospective and observational studies. Only one study provided a direct comparison between children with and without DS who were using long term NIV (83). Additional work is needed to understand potential challenges around establishing long-term NIV use in children with Down Syndrome, clinical outcomes, impacts on quality of life and facial growth.

An alternative in patients with CPAP mask intolerance may be high flow nasal cannula therapy (84). Amaddeo *et al*. studied HFNC in 8 patients who were intolerant to CPAP, of whom six had DS. Three out of the 6 patients with Down Syndrome were successfully managed with HFNC, the other patients did not tolerate HFNC well (85). Advances such as custom-made masks for NIV may improve tolerance for children for whom mask ventilation is the only available treatment option (86).

#### Medication

Anti-inflammatory medications have been shown to be an effective treatment for mild OSA in otherwise healthy children (45, 87–89). Kheirandish-Gozal et al. reported significant reduction in the severity of OSA in children with the use of intranasal budesonide (87) and oral montelukast, a leukotriene inhibitor (88). Goldbart et al. reported that montelukast could effectively improve polysomnographic measures and symptoms for mild OSA in children aged 2–10 years. A retrospective study conducted by Kheirandish-Gozal et al. (90) showed beneficial effects using a combination of intranasal corticosteroids and oral montelukast in more than 80% of children aged 2–14 years with mild OSA after 3 months of treatment. However, these studies all excluded children with genetic disorders such as DS and the efficacy in children with DS and mild OSA has not been extensively investigated.

Yu et al. examined the polysomnographic changes of children with DS and mild OSA treated with medication (91). In a retrospective cohort, medication therapy did not significantly improve polysomnographic measures in children with DS and mild OSA. Prospective studies are required to investigate this further.

#### Orthodontic appliances

Orthodontic assessment may be of benefit to some children with DS who have a greater incidence of orthodontic factors such as cross-bite and narrow palate associated with their distinct orofacial features. Improved SDB has been reported in a case study of a 15 year old with DS and severe OSA as well as teeth crowding and posterior crossbite who had no adenotonsillar enlargement and could not tolerate CPAP (92) who underwent rapid palatal expansion (RPE). RPE causes the separation of the midpalatal suture, thus causing expansion of the nasal floor and airway and increasing the oral cavity volume. Therefore, RPE can be a viable treatment option for both OSA and malocclusion in patients with DS, primarily because it is effective in resolving orthodontic problems without the need for patient compliance. In a second case study, significant improvements in the obstructive apnea-hypopnea index, sleep efficiency, and arousal index following RPE (93).

## Gastrointestinal disorders affecting respiratory health

Feeding problems and gastrointestinal (GI) disorders are prevalent in children with DS. The connection between GI and respiratory health is critically important as GI dysfunction can significantly affect respiratory health, leading to chronic respiratory conditions, frequent infections, and poor overall quality of life. Understanding this relationship is key to improving care and outcomes for children with DS.

### Interactions between gastrointestinal disorders and respiratory health

The interplay between gastrointestinal and respiratory health in children with DS is complex and multifactorial. The following key factors contribute to the high prevalence of GI-related respiratory complications in this population.

#### Dysphagia and aspiration

Dysphagia is highly prevalent in children with DS and is often associated with silent aspiration into the lower airways (94). The underlying cause of dysphagia in DS is multifactorial, including hypotonia affecting the muscles involved in swallowing, delayed motor development, and coordination deficits (95).

Studies such as those by Narawane et al. (96) and Jackson et al. (94) have demonstrated the high prevalence of dysphagia in children with DS. Narawane et al. evaluated 127 infants with DS (mean age 4 months) using videofluoroscopy, revealing that 89.8% had oral dysphagia and 72.4% had pharyngeal dysphagia, with 31.5% showing tracheal penetration of thin liquids, often silently. Similarly, Jackson et al. found that 44.2% of 138 children with DS (mean age 2 years) experienced aspiration, with 56.3% showing pharyngeal dysphagia.

Chronic aspiration can lead to wheezing (97) chronic cough, or recurrent lower respiratory tract infections (98), potentially resulting in bronchiectasis, impaired lung function, and pulmonary hypertension (99). Given the prevalence of swallowing dysfunction in children with DS, early identification and intervention are critical to prevent long term respiratory sequelae. The timing of such assessments is still part of an ongoing debate. Some experts advocate for routine screening soon after birth or when early symptoms appear, to prevent long term damage from aspiration (94). On the other hand, some argue that assessments should be symptom-driven based on observed feeding difficulties or evidence of recurrent respiratory infections, to avoid unnecessary interventions (100). The role of videofluoroscopic swallow studies vs. clinical assessments in detecting silent aspiration is also a matter of debate. Videofluoroscopy may not be available in all healthcare settings and is associated with radiation exposure.

#### Gastroesophageal reflux disease (GORD)

GORD is highly prevalent in children with DS, likely due to reduced muscle tone in the lower oesophageal sphincter along with developmental abnormalities in the enteric nervous system, which disrupt normal gastrointestinal motility (101). In these children, GORD often presents atypically and can be largely asymptomatic.

GORD is also closely linked to obstructive sleep apnoea (OSA). In a study by Trucco et al. (82), GORD was detected in 47% of children with DS who experienced OSA, indicating a significant overlap between the two conditions. There remains disparity in the approach to managing GORD in children with DS. Some centres advocate aggressive management with early use of proton pump inhibitors and surgical intervention such as fundoplication to prevent long term lung damage. Others argue that PPIs should be used in children with DS until they achieve improved muscle tone and independent walking (102). A more conservative approach has also been suggested with an emphasis on dietary changes, positioning therapy, thickened feeds and only cautious use of PPIs, which and increase risk of infections and nutrient malabsorption (100). The debate highlights the need for individualised treatment plans that are based on severity of symptoms, frequency of aspiration, response to initial therapies and impact on respiratory health.

#### Congenital gastrointestinal malformations

Gastrointestinal malformations are reported in 3%–13% of children with DS (103), with conditions such as oesophageal atresia and tracheoesophageal fistula (TOF) being notably more common than in the general population. These malformations often require early surgical intervention but can result in long-term respiratory complications, such as cough, bronchitis, and recurrent respiratory infections, especially when post-repair complications like strictures or recurrent fistulas develop (104). Oesophageal atresia alone has a prevalence of 0.4% in children with DS (19), and studies show that nearly two-thirds of children who undergo oesophageal repair continue to experience respiratory symptoms for up to five years postoperatively (26).

## Immunodeficiency and immune dysregulation

Since the 1970s it has been recognised that the immune system of individuals with DS behave differently to those without DS (105, 106). Children and adults with DS are at increased risk of infections (particularly of the respiratory tract), autoimmune disorders (such as hypothyroidism and coeliac disease), as well as haematological malignancies (such as acute lymphoblastic leukaemia and transient myeloproliferative disease), all of which suggest a level of immunodeficiency and immune dysregulation (105, 107). Chromosome 21 itself encodes for several important immune regulatory genes, such as those responsible for neutrophil function (SOD1, ITGB2), interferon receptors (IFNAR1 and IFNAR2) and autoimmune regulator (AIRE) genes. It is speculated that gene overexpression could be a reason behind the altered and dysregulated immune system seen on multiple levels in DS, however this is not fully understood (108, 109).

### More frequent and severe infections

Children and adults with DS are more susceptible to infections compared to those without DS, particularly infections of the respiratory tract, periodontal tissue and skin (107). Bacterial infections in children with DS are associated with a substantially higher mortality rate compared to the non-DS population. Garrison et al. calculated the risk of mortality from sepsis and showed children with DS had a 30% higher risk of fatality secondary to sepsis compared to healthy controls (mortality rate ratio 1.3, 95% confidence intervals 1.06–1.59), even after controlling for pathogen types and host co-morbidities (110). The increased risk of complications and mortality associated with infections continues into adulthood (108).

Lower respiratory tract infections are recognised as the most common cause for acute hospital admission for children with DS, and the most common cause to need intensive care admission. A retrospective review of 232 admissions of children with DS done by Hilton et al. showed 54% of admissions were for respiratory issues. Length of hospital stay and admission cost for respiratory conditions were two to three times greater compared to non-DS controls, even when adjusted for congenital heart disease (111). With more severe and prolonged courses of respiratory disease, there is an increased risk of progression to acute lung injury and acute respiratory distress syndrome compared to non-DS controls, all of which contribute to protracted recovery and added morbidity after the acute phase of infection (112).

DS is now recognised as an independent risk factor for severe RSV lower respiratory tract infections in children, and this vulnerability is increased with additional medical co-morbidities such as congenital heart disease and prematurity (113). A large international meta-analysis showed that children with DS infected with RSV have an 8.7 times high risk of hospital admission, are 2.7 times more likely for ICU admission, and have a 9 times higher mortality risk compared to children without DS (114).

### Altered immune parameters

Individuals with DS exhibit abnormal immune laboratory parameters, in both the cellular and humoral arms, however, the clinical significance of these abnormalities remains unclear. Those with DS have a lower number of circulating T and B lymphocytes from birth, with almost 90% of DS children exhibiting T and B cell subsets lower than the 10th percentile, and 60% below the 5th centile of normal (115). While lymphocyte populations are expected to have a large expansion in infancy, this is not seen in DS (115, 116). With time, the T lymphocyte counts are expected to gradually approach normal ranges, but the individual cells may not have the same functionality compared to healthy controls. In contrast, B lymphocyte counts of all stages remain low compared to the normal population (106, 115). Interestingly, there is no clear association between low *T* and B cell counts with clinical conditions such as recurrent infection (106, 107).

Naive *T* cell populations are usually mild or moderately reduced in DS, as well as lower *T* cell receptor excision circles (TRECs, which are byproducts of *T* cell receptor recombination), thus reflecting inefficient *T* cell differentiation in the thymus. A smaller thymic size and abnormal thymic structure has been suggested as a contributing cause to thymic dysfunction although this is likely to be multifactorial (106). Lymphocyte function tests (where T and B lymphocyte proliferation are measured in response to mitogens) are often low in children and adults with DS (105, 106), while there are also reports of increased apoptosis markers found on DS T lymphocytes (117). Aside from lymphocyte abnormalities, defective neutrophil chemotaxis, as well as reduced natural killer (NK) cell activity have also been reported, while studies have not been consistent in reporting defective oxidative burst responses to pathogens (107, 108).

In children with Down Syndrome, humoral immunity shows variability between individuals and changes as they age, with increased serum IgG and IgA levels after age 5 and decreased IgM during adolescence (106). Specific antibody responses to vaccinations were also consistently lower than those without DS (including for pneumococcus, Hepatitis B, tetanus and polio vaccines). Although this was often still at protective levels and does respond to vaccine boosting, this protection may not be as long-lasting as those without DS (107, 108). There is also a deficiency of complement proteins (108).

There is increasing evidence that there is altered toll-like receptor (TLR) signalling which triggers elevated cytokine levels (such as IL-1alpha, IL4, IL6, IL13, TNF and interferon-alpha), which has been seen in healthy individuals with DS even in the absence of infection. This “cytokinopathy” is likely to contribute to the higher incidence of autoinflammatory conditions and para-infectious inflammatory sequelae accompanying infection despite fewer numbers of differentiated circulating lymphocytes and can contribute to worsened infection outcomes (107–109). Despite this, allergy is not a dominant issue among DS children: DS children who report symptoms of chronic rhinitis and reactive airways disease generally have low levels of total IgE and fewer positive skin prick tests compared to the non-DS population (106, 107).

### Non-immune factors that cause increased infection susceptibility

While abnormal immune responses are likely to play a significant role in explaining why children with DS have poorer infection-related outcomes, this is likely to be multi-factorial. Children with DS often have anatomical co-morbidities that increase infection risk. Congenital external ear canal stenosis predisposes to chronic otitis media, and airway abnormalities such as laryngomalacia and tracheomalacia, impair secretion clearance and facilitate infections (103). In addition to this, medical co-morbidities such as congenital heart disease, gastro-oesophageal reflux and reactive airway disease may also contribute to the development of infection and slow recovery. Secondary immunodeficiency due to metabolic and nutritional factors particularly zinc deficiency, has been postulated but not proven (107).

A multidisciplinary approach to investigation and management of recurrent, prolonged or severe infections would therefore be indicated for children with DS. Further research is necessary to better understand immune pathology seen in DS, and how to tailor management strategies and medication for this group of patients.

## Current gaps and future developments

Table 1 summarises the respiratory and airway complications in children with DS described in this article, including clinical implications and management strategies; however, despite advancements in our understanding of respiratory health in children with DS, gaps in our knowledge remain. There is a lack of large, longitudinal studies examining the long-term outcomes of children with DS who have GI disorders and respiratory complications. Understanding how early interventions such as the treatment of GORD, dysphagia management, or surgical repair of GI malformations impact long-term respiratory health could help guide clinical decision-making for future cohorts of children with DS.

**Table 1:** Summary of respiratory and airway complications in children with DS, including clinical implications and management strategies.

Complication	Pathophysiology	Clinical implications	Management strategies
Obstructive sleep apnoea	Hypotonia, macroglossia, adenotonsillar hypertrophy, and midface hypoplasia cause airway collapse	Daytime fatigue, behavioural problems, and cardiovascular strain	NIV, medical treatments, adenotonsillectomy, orthodontic appliances, Hypoglossal nerve stimulation
Recurrent lower respiratory tract infections	Impaired immune function and anatomical abnormalities, poor airway clearance	Chronic productive cough, recurrent exacerbations, progressive lung function decline	Prompt treatment of infections, prophylactic antibiotics, airway clearance techniques, chest physiotherapy
Congenital airway anomalies	Laryngomalacia, tracheomalacia, subglottic stenosis, tracheal bronchus and narrow upper airways	Stridor, noisy breathing, recurrent croup, and risk of critical airway obstruction	ENT evaluation, surgical interventions (e.g., tracheoplasty), and monitoring during illness
Pulmonary hypertension	Often secondary to OSA, congenital heart disease, or chronic hypoxia	Risk of right heart failure, reduced exercise tolerance	Treat underlying causes (OSA or heart defects), supplemental oxygen, pulmonary vasodilators
Gastroesophageal reflux and aspiration	Hypotonia and swallowing dysfunction lead to recurrent aspiration	Chronic wet cough, recurrent Lower respiratory tract infections and faltering growth	Medical treatments, Speech and language therapy, Video fluoroscopy, thickened feeds, gastrostomy tube placement

There is also emerging evidence suggesting an ongoing relationship between the gut microbiome and respiratory health known as the gut-lung axis (118). In children with DS, the microbiome is often dysregulated due to a combination of genetic factors, frequent use of antibiotics, and underlying GORD. An imbalance in gut microbiota, known as dysbiosis, may contribute to systemic inflammation and increase susceptibility to respiratory conditions such as asthma, chronic bronchitis, and recurrent infections, however, little is known about the specific microbiome changes in DS and how they influence GI and respiratory health. The potential for microbiome-targeted therapies, such as probiotics or dietary modifications, is an area of active investigation to improve both GI and respiratory outcomes in these children.

The relationship between gastrointestinal disorders and respiratory health in children with DS is complex and multifactorial. Future developments in the care of children with Down Syndrome may therefore focus on innovative diagnostic technologies, targeted therapies, and integrated care models. As genetic understanding advances, targeted therapies, including gene therapies, may address GI and respiratory dysfunction by correcting underlying neuromuscular abnormalities. Multidisciplinary care models are vitally important and should be central to the management of these children, integrating multiple specialties and disciplines for more coordinated care.

Hypoglossal nerve stimulation (HNS) is a novel therapy that has shown efficacy in treating adults with OSA (119, 120). Given the lower success rates with surgical and NIV treatments compared with the typical population, it has been proposed that HNS may be beneficial for children with DS and OSA.

A hypoglossal nerve stimulator comprises of an implantable pulse generator (IPG), a pressure sensor to detect breathing, and stimulation leads connected to the sublingual nerve (121). The pressure sensor monitors chest wall motion, allowing the IPG to signal the end of expiration and the beginning of inspiration. The stimulation is subsequently delivered to the hypoglossal nerve through the IPG, and the stimulation leads can specifically activate certain branches of the hypoglossal nerve, which enhances the stiffness and protrusion of the tongue (122). Tongue protrusion expands the cross-sectional dimensions of the airway, consequently facilitating the patient's airway; and thus, preventing airway collapse (123).

In 2016, Diercks *et al*. reported the first case of HNS treatment for a 14-year-old boy, whose AHI dropped from 48.5 to 3.4 events/h (124). A review by Liu et al. identified nine articles that included 106 patients including a Phase 1 clinical trial of hypoglossal nerve implants in adolescents with Down Syndrome with persistent severe OSA after adenotonsillectomy and who were not able to tolerate CPAP (125). All the studies showed that patients receiving hypoglossal nerve stimulation experienced a significant decrease in apnoea-hypopnea index (at least 50%). The pooled AHI was significantly lower in patients following treatment (mean AHI reduction 17.43 events/h, 95% confidence interval 13.98–20.88 events/h, *P* < 0.001). The pooled OSA-18 (a validated, disease-specific quality of life instrument for OSA) was significantly decreased in 88 patients after treatment (mean OSA-18 reduction 1.67, 95% confidence interval 1.27–2.08, *P* < 0.001). Most studies had relatively short patient follow-up periods, with the most extended follow-up being 44–58 months. In addition to this review of the respiratory outcomes, a study of neurocognition and behaviour in nine adolescents treated with HNS found that these participants had better neurocognitive and behavioural scores after 6.5 months of treatment for an average of 15.2 ± 3.4 h per day (126).

The most common complications reported are pain or discomfort in the tongue or mouth (7.3%). Notably, three studies documented the occurrence of serious adverse events, with overall incidences of readmission 10.1%, and 5.9% re-operation rates. These adverse events were primarily caused by device displacement, infection, device migration, and poor postoperative pain control.

The evidence from the literature so far indicates that HNS can significantly reduce the AHI and improve the quality of life of adolescents with DS and can be considered as a potential alternative treatment for OSA. More studies are required to fully demonstrate the long-term efficacy of HNS, potential complications, adverse events, comfort, and cost-effectiveness. Direct comparison with CPAP and upper airway surgery, variation in voltage stimulation intensity and co-morbidities such as obesity are also important factors for future investigation.
